# Nucleic acid testing of SARS-CoV-2: A review of current methods, challenges, and prospects

**DOI:** 10.3389/fmicb.2022.1074289

**Published:** 2022-12-09

**Authors:** Yuanshou Zhu, Meng Zhang, Zhijun Jie, Shengce Tao

**Affiliations:** ^1^Key Laboratory of Systems Biomedicine (Ministry of Education), Shanghai Center for Systems Biomedicine, Shanghai Jiao Tong University, Shanghai, China; ^2^School of Biomedical Engineering, Shanghai Jiao Tong University, Shanghai, China; ^3^Department of Pulmonary and Critical Care Medicine, Shanghai Fifth People’s Hospital, Fudan University, Shanghai, China; ^4^Center of Community-Based Health Research, Fudan University, Shanghai, China

**Keywords:** SARS-CoV-2, nucleic acid testing, biosensors, CRISPR-based diagnostic, COVID-19 variants

## Abstract

Coronavirus disease 2019 (COVID-19) is caused by severe acute respiratory syndrome coronavirus 2 (SARS-CoV-2) and has brought a huge threat to public health and the global economy. Rapid identification and isolation of SARS-CoV-2-infected individuals are regarded as one of the most effective measures to control the pandemic. Because of its high sensitivity and specificity, nucleic acid testing has become the major method of SARS-CoV-2 detection. A deep understanding of different diagnosis methods for COVID-19 could help researchers make an optimal choice in detecting COVID-19 at different symptom stages. In this review, we summarize and evaluate the latest developments in current nucleic acid detection methods for SARS-CoV-2. In particular, we discuss biosensors and CRISPR-based diagnostic systems and their characteristics and challenges. Furthermore, the emerging COVID-19 variants and their impact on SARS-CoV-2 diagnosis are systematically introduced and discussed. Considering the disease dynamics, we also recommend optional diagnostic tests for different symptom stages. From sample preparation to results readout, we conclude by pointing out the pain points and future directions of COVID-19 detection.

## Introduction

Coronavirus disease 2019 (COVID-19), caused by severe acute respiratory syndrome coronavirus 2 (SARS-CoV-2), is a highly contagious and lethal disease. As of July 7, 2022, there have been over 548 million diagnosed cases and 6.3 million deaths.^1^ Rapid and accurate diagnosis and the immediate isolation of infected individuals are crucial for controlling outbreaks. Serological tests are usually not able to detect early infections due to late seroconversion after infection, so they usually serve as complementary detection methods. Because of its high sensitivity and specificity, nucleic acid testing (NAT) is regarded as the first choice for SARS-CoV-2 detection. The basic requirements for NAT include high accuracy, high specificity, high sensitivity, high speed, and low cost. Furthermore, the SARS-CoV-2 outbreak may coincide with seasonal influenza, which causes similar symptoms. The visits of influenza patients to hospitals also contributed to the increasing spread of SARS-CoV-2 infection (Wolfel et al., 2020), which puts forward higher requirements for detection, including detection throughput, multiplicity, and portability.

**FIGURE 1:**
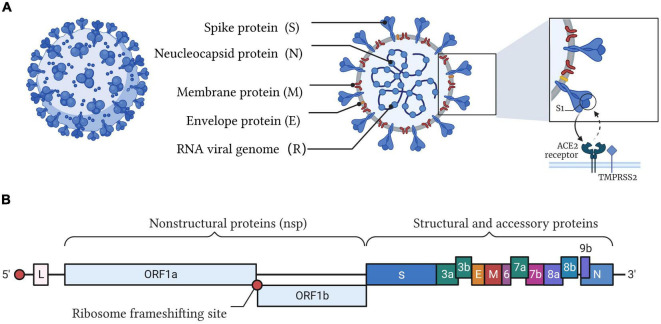
Illustration of the SARS-CoV-2 structure and genome. **(A)** SARS-CoV-2 belongs to the cluster of β-coronaviruses with spherically enveloped virions. Envelope glycoproteins such as spike protein (S), envelope protein (E), and membrane protein (M) are embedded in a lipid bilayer envelope. Nucleocapsid protein (N) coupled with single-stranded positive-sense viral RNA is inside the envelope. Through the interaction between the receptor-binding domain (RBD) of the spike protein and angiotensin converting enzyme 2 (ACE2), SARS-CoV-2 invades host cells. **(B)** The genome of SARS-CoV-2 encodes four major structural proteins (S, E, M, N), 16 non-structural proteins (nsp), and 8 accessory proteins.

Severe acute respiratory syndrome coronavirus 2 is an enveloped single-stranded RNA virus that belongs to the genus β-coronavirus (Harvey et al., 2021). The genome of SARS-CoV-2 is approximately 30 kb and encodes four major structural proteins [spike (S), envelope (E), membrane (M), and nucleocapsid (N)], 16 non-structural proteins, and 8 accessory proteins (Figure 1). It has been reported that SARS-CoV-2 evolves at a rate of approximately 1.1 × 10^–3^ substitutions per site per year, which corresponds to nearly one substitution every ∼11 days (Rambaut et al., 2020). For current high-risk variants of SARS-CoV-2, the WHO prompted their classification as Variants of Concern (VOCs) and Variants of Interest (VOIs) (World Health Organization, 2022a). The emergence of novel variants of SARS-CoV-2, especially for Omicron variants, further highlights the challenges of diagnosis and treatment when we are facing this pandemic. To further group these variants, many research organizations and WHO are working on the identification and classification of SARS-CoV-2. Although various nomenclature, such as country and Greek names, have been tried, a greater grouping, such as lineages and clades, is indispensable. One of the most accepted proposals is put up by GISAID, which identified the variants into eight global clades (S, O, L, V, G, GH, GR, and GV) (Global Initiative on Sharing Avian Influenza Data, 2022). GISAID provides open access to genomic data of SARS-CoV-2, and based on the data, the ARTIC Network provides a common resource of PCR primer sequences and recommendations for amplifying SARS-CoV-2 genomes (Artic network, 2022). Real-time tracking later was used for tracking SARS-CoV-2. As of June 2021, Nextstrain has identified 13 major clades (19A–B, 20A–20J and 21A) (Hadfield et al., 2018; Nextstrain, 2022). The other well-known nomenclature is PANGOLIN proposed by Rambaut et al. in the Phylogenetic Assignment of Named Global Outbreak Lineages (PANGOLIN) (Rambaut et al., 2020; Github, 2022; World Health Organization, 2022b). As of August 2021, 1,340 lineages had been designated (Rambaut et al., 2021; Cov-lineages, 2022). Therefore, a rapid (high detection throughput and less time-consuming), accurate (high sensitivity, specificity, and multiplicity), and low-cost POCT (point-of-care testing) method is in high demand for the timely identification of positive cases and effective tracing of potential SARS-CoV-2-infected individuals.

**FIGURE 2:**
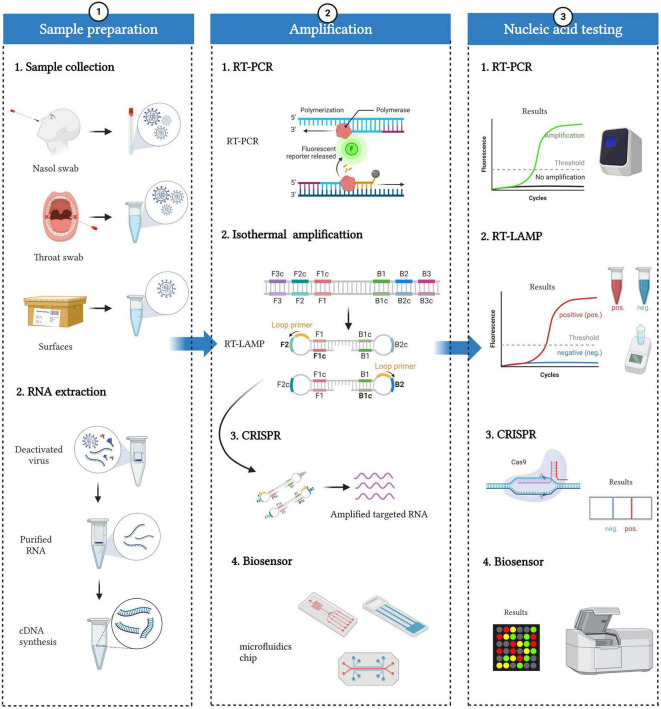
Workflow of SARS-CoV-2 nucleic acid detection. The whole detection process can be divided into three parts: sample preparation, amplification, and nucleic acid testing. Sample preparation included sample collection and RNA extraction. Specimens mainly come from people (such as nasal swabs and throat swabs) and the environment (such as the surface of packages). RNA can be extracted from deactivated virus, and then cDNAs are synthesized based on reverse transcriptase. Various nucleic acid amplification methods, including RT-PCR, isothermal amplification, CRISPR, and biosensors, can be applied to amplify and detect cDNAs.

This review aims to present a comprehensive view of different nucleic acid testing methods for COVID-19 and help researchers make an optimal choice in detecting COVID-19 at different symptom stages. Here, we first outline the general workflow of SARS-CoV-2 detection and describe it in three steps: sample preparation, amplification, and nucleic acid testing (Figure 2). Then, we systematically summarize the current nucleic acid methods by emphasizing their pros and cons (Table 2). The unique selling points of this review are to introduce emerging biosensors and CRISPR-based diagnostic systems and specifically discuss the impact of COVID-19 variants on SARS-CoV-2 diagnosis. In view of the disease dynamics, we suggest choosing suitable diagnostic methods for different symptom stages. Moreover, the reasons related to false-negative and false-positive results in practice are also explained. Finally, we point out the pain points and future directions for the development of SARS-CoV-2 detection methods.

## Workflow of severe acute respiratory syndrome coronavirus 2 detection

The diagnosis of COVID-19 mainly consists of three parts: sample preparation, amplification, and nucleic acid testing (Figure 2).

The sample preparation included sample collection and RNA extraction. For sample collection, specimens mainly come from people (such as nasal swabs and throat swabs) and the environment (such as the surface of packages). It has been reported that diverse samples from different parts of people show a great difference in positive rates. For example, Wang et al. (2020) collected 1,070 samples from 205 COVID-19 patients and found that bronchoalveolar perfusion fluid samples (93%), sputum (72%), and nasal swabs (63%) were the three sources with the highest positive rates. In practice, the World Health Organization (WHO) recommends sample collection from nasopharyngeal (NP) and oropharyngeal (OP) swabs and from the lower respiratory tract of patients on mechanical ventilation. NP swabs in the detection of SARS-CoV-2 are superior to OP swabs, possibly because of the higher virus load in the nose (Tu et al., 2020; Wyllie et al., 2020; Lee et al., 2021). Apart from people, the environment, such as the surface of packages and wastewater, is also critical with an increasing chance of spreading infection in large-scale outbreaks. For example, Zhang et al. (2022) found that the increased confirmed cases on campus have a positive correlation with positive environmental samples. In sewage treatment plants and densely populated cities, wastewater is also related to active confirmed COVID-19 cases (Tandukar et al., 2022).

After sample collection, there are two main routes for treatment: RNA extraction for amplification and direct amplification without RNA extraction. The former would be more accurate with purified nucleic acids, which are often applied in clinical detection. RNA was extracted from deactivated samples, and cDNAs were synthesized based on reverse transcriptase for detection. Solid-phase extraction (SPE), relying on silica membranes or beads, filter papers, or polymer resins, is widely applied in clinical RNA purification with high accuracy. Incorporating SPE with a microfluidic chip makes nucleic acid testing more appropriate for patients with a high risk of viral transmission (Soares R. et al., 2021). Undoubtedly, detection performance can be greatly affected by the efficiency of different RNA extraction methods. Currently, several methods are used to extract RNA from samples, mainly including anion-exchange resin, magnetic bead-based methods, and guanidinium thiocyanate-phenol-chloroform extractions. Ambrosi et al. (2021) compared the efficiency of three different commercial kits (based on silica-gel affinity columns) and an in-house extraction protocol (based on EXTRAzol) for SARS-CoV-2 detection. They found that the Qiamp DSP Virus Spin kit (Qiagen, Cat. 61704) achieved the highest extraction efficiency, followed by the Viral Nucleic Acid (DNA/RNA) Extraction Kit I (Fisher Molecular Biology, Cat. DR-003), Total RNA Purification Kit (Norgen, Cat. # 17200), and EXTRAzol (BLIRT S.A., Cat. EM30-100). Therefore, caution should be exercised for the detection of SARS-CoV-2 with EXTRAzol, since in the presence of low or very low viral loads, it can go undetected. Nevertheless, purification will cause some loss of RNA and then decrease the sensitivity of detection. In contrast, direct amplification can avoid RNA loss and save considerable operation time. Some companies, such as Sansure Biotech (Banko et al., 2021; Wen et al., 2021) and Vazyme Biotech (Bruce et al., 2020; Qin et al., 2021), have launched lysis buffers for samples for direct amplification. Of course, unextracted samples may lead to the impurity of nucleic acids, inhibition of the reaction, and reduced sensitivity and accuracy. Therefore, nucleic acid extraction can be selected according to specific scenes and detection requirements.

Finally, various nucleic acid testing (NAT) methods, including sequencing, RT-PCR (reverse transcription polymerase chain reaction), isothermal amplification, CRISPR (clustered regularly interspaced short palindromic repeat), and biosensors, have been developed for SARS-CoV-2 detection. None of them is perfect, and different detection methods have their own strengths and weaknesses. We will introduce and evaluate these methods in detail below.

## Sequencing technology

High-throughput sequencing (HTS), also known as next-generation sequencing (NGS), can capture the pathogen from complex samples and analyze the complete information of its genome in detail, which is beneficial to the discovery of unknown pathogens. During the COVID-19 pandemic, NGS has been widely used in the comprehensive characterization and analysis of SARS-CoV-2 and surveillance of new variants. Chinese scientists were the first to extract viral RNA from bronchoalveolar lavage fluid (BALF) and complete the sequencing of SARS-COV-2 genomes and reported the emergence of this new virus (Wu et al., 2020). Apart from BALF, nasopharyngeal (NP) swabs are also used as an appropriate source of sequencing samples. Through metagenomics NGS (mNGS), we can also acquire information about the composition of the respiratory microbiome, SARS-CoV-2 coinfection, and the presence of other organisms that may influence infection progression. One of the greatest strengths of NGS is to screen and identify viral pathogens without prior knowledge of the pathogen, while with high sensitivity and specificity. Currently, some commercial applications are under development, including BioCat, Arbor Biosciences and Swift testing based on NGS (Addetia et al., 2020).

To further improve the sample processing speed, portability, and read length, nanopore third-generation sequencing (NTS) has emerged. SARS-CoV-2 was sequenced through nanopore technology and the sequence-independent single primer amplification (SISPA) method (Chan J. F. et al., 2020), which did not require chemical labeling or PCR amplification of samples, thereby improving detection efficiency. Viehweger et al. (2019) used a direct RNA sequencing (DRS) method based on nanopores to detect viral RNA produced in SARS-COV-2-infected cells. They could map the longest (∼26 kb) continuous read to the viral reference genome, bypassing RNA reverse transcription and amplification to detect methylation sites in viral RNA. The specificity and sensitivity of NTS for SARS-CoV-2 detection are much higher than those of RT-PCR detection. However, it is necessary to strengthen research on improving sequencing accuracy and reducing background interference in the future.

Apart from the identification of pathogens, HTS has been applied for environmental and food safety monitoring, human and plant genome sequencing, and antibiotic resistance detection (Liu Y. X. et al., 2021; Maina et al., 2021). However, it is difficult to avoid the high cost, time consumption and need for highly professionals. Therefore, HTS is often used for unknown pathogen identification rather than routine nucleic acid testing.

## Reverse transcription polymerase chain reaction

Reverse transcription polymerase chain reaction (RT-PCR) is currently regarded as the gold standard by the WHO for the diagnosis of COVID-19 (Mizumoto et al., 2020; Soares R. R. G. et al., 2021). Based on reverse transcriptase synthesizing complementary DNA (cDNA) from the viral RNA template, RT-PCR could generate double-stranded DNA, which can be detected using a TaqMan probe or DNA-embedded dye. The cyclic threshold (Ct) value could be used to evaluate the virus load. A Ct value below 35 is considered COVID-19 positive, and a Ct value above 35 is considered COVID-19 negative (Kampf et al., 2021). At present, the target genes detected by RT-PCR mainly include ORF1ab, E, N, and non-structural RNA-dependent RNA polymerase (RdRp), which are conserved or highly expressed (Kevadiya et al., 2021). Many commercial kits based on RT-PCR have been developed. For example, the United States has approved an Emergency Use Authorization (EUA) for RT-PCR kits for emergency use of diagnosing COVID-19, including Invitrogen SuperScript IV (Thermo Fisher, Waltham, MA, USA), Xpert Xpress SARS-CoV-2 Test (Cepheid, Sunnyvale, CA, USA), BioFire Respiratory Panel 2.1-EZ (BioFire Diagnostics, Salt Lake City, UT, USA), and other kits (Food and Drug Administration, 2022). The kits mentioned above are based on a one-step amplification method, reducing the risk of cross-contamination and artificial fault. In contrast, the two-step assay could improve the sensitivity and level of detection; however, there are some limitations, e.g., time consumption, aerosol contamination and requirements for optimizing parameters, that need to be solved.

It is necessary to note that the application of RT-PCR is limited by viral load. RT-PCR is not sensitive to excessively low viral loads in the very early stage of COVID-19 infection. In comparison, digital PCR (dPCR) can detect mutations as low as one copy, enabling the identification of SARS-CoV-2 in the very early infection period. Digital PCR, based on the principles of limited dilution, end-point PCR and Poisson statistics, has a broader dynamic range without external interference and robustness to variations in PCR efficiency. Additionally, an independent reaction system of dPCR can quantify the initial sample absolutely, which is much more beneficial for clinical analysis. Furthermore, droplet digital PCR (ddPCR) based on water-in-oil droplets displays greater superiority in clinical diagnosis along with a much higher dynamic detection range and accuracy than dPCR (Dong et al., 2021). However, ddPCR relies on much more expensive instruments and reagents, limiting its use in mass nucleic acid screening of SARS-CoV-2.

To further increase the detection efficiency and accuracy of PCR in the detection of multiple variants, multiplex PCR (mPCR) was developed, which can simultaneously amplify multiple target sequences in a single reaction. mPCR has been widely used in identifying mutations of SARS-CoV-2 and variants with the advantages of time savings, high sensitivity and accuracy, and high multiplicity. mPCR is also suitable for distinguishing between SARS-CoV-2 and other respiratory viruses. Using QIAstat-Dx Respiratory Panel V2 (Qiagen, Hilden, Germany), Bouzid et al. (2021) detected 22 respiratory viruses and bacteria within 1 h, which promotes the management of patients with similar respiratory symptoms.

In general, RT-PCR is suitable for large-scale nucleic acid screening and has played an important role in the COVID-19 pandemic. Nevertheless, RT-PCR also has some limitations, such as a long reaction time, sophisticated thermal cycling instruments, and skilled operators. With the emergence of isothermal amplification, microfluidics and CRISPR technologies, these problems have been solved to some extent.

## Isothermal amplification

Although RT-PCR has been widely adopted in the diagnosis of COVID-19, it has some limitations. As opposed to RT-PCR, isothermal-based amplification, such as reverse transcription loop-medicated isothermal amplification (RT-LAMP) and reverse transcription recombinase polymerase amplification (RT-RPA), can be carried out at a constant temperature and only requires minimal energy input. This feature enables them to be developed into potential point-of-care testing (POCT) methods.

### Reverse transcription loop-medicated isothermal amplification

Reverse transcription loop-medicated isothermal amplification is currently one of the most common isothermal amplification methods, which was first reported in 2000 by Notomi et al. and can achieve 10^9^∼10^10^-fold amplification within 15∼60 min at 60∼65°C (Notomi et al., 2000). Two pairs of primers, known as inner primers and outer primers, are designed to recognize six specific regions of the target genes. To accelerate amplification, two loop primers are often added simultaneously to amplify the loop region of intermediate products. Due to its high sensitivity, specificity and convenience, RT-LAMP has been extensively applied in pathogen detection.

During the COVID-19 pandemic, a series of isothermal detection methods based on RT-LAMP have been developed, which can be divided into three categories. In the beginning of the outbreak of COVID-19, many researchers adopted conventional colorimetric RT-LAMP to establish similar SARS-CoV-2 detection methods in tubes and achieved good results (Dao Thi et al., 2020; Ludwig et al., 2021). If the sample is positive, the color of the reaction mixture will change from pink to yellow or intermediate orange. Second, to improve portability and multiplicity, Zhu et al. (2020) devised multiplex RT-LAMP (mRT-LAMP) coupled with a nanoparticle-based lateral flow biosensor (LFB) assay (mRT-LAMP-LFB) for COVID-19 diagnosis. They simultaneously amplified the ORF1ab and N genes of SARS-CoV-2 in a single-tube reaction and detected results with LFB. In contrast, Yang M. et al. (2021) applied a four-channel microfluidic chip to combine ultrasensitive RT-LAMP for SARS-CoV-2 detection, which can largely avoid contamination from aerosols. Third, some emerging technologies, including clustered regularly interspaced short palindromic repeat (CRISPR) and Argonaute (Ago), are also integrated with RT-LAMP for SARS-CoV-2 detection. For example, Broughton et al. (2020) reported a CRISPR-Cas12a-based lateral flow assay to detect SARS-CoV-2 from swab RNA extracts, which was called SARS-CoV-2 DNA Endonuclease-Targeted CRISPR Trans Reporter (DETECTR). Joung et al. (2020) applied Cas12b to establish an integrated viral detection platform named STOPCovid (SHERLOCK testing in one pot). Xun et al. (2021) developed a rapid scalable and portable testing (SPOT) system, which comprises one-pot RT-LAMP followed by PfAgo (Argonaute protein from Pyrococcus furiosus)-based target sequence detection. Ye et al. (2022) also reported a multiplex Argonaute (Ago)-based nucleic acid detection system (MULAN) to simultaneously detect SARS-CoV-2 and influenza viruses. Finally, molecular beacons were also adapted to detect RT-LAMP products of SARS-CoV-2 so that Sherrill-Mix et al. (2021) constructed the LAMP-BEAC platform.

Therefore, as a general isothermal amplification method, RT-LAMP can be integrated with different technologies to develop high-efficiency detection platforms. However, cross-reactivity can be an issue because of a higher number of primers. If we intend to realize multiplex detection in one tube, the design and optimization of the primers can be tedious and complex. Meanwhile, aerosol contamination is another common problem when we perform RT-LAMP. Hence, combining the strengths of other technologies is the future direction of RT-LAMP.

### Reverse transcription recombinase polymerase amplification

Reverse transcription recombinase polymerase amplification (RT-RPA) is another promising isothermal amplification method, which was first reported in 2006 by Piepenburg et al. and can achieve ∼10^12^-fold amplification within 20~40 min at 37~42°C (Piepenburg et al., 2006). Three key proteins, including recombinase (UvsX), strand-displacing polymerase (Bsu), and single-strand binding (SSB) protein (gp32), and other accessory proteins are in collaboration with two specific primers (between 30 and 36 bp) to amplify the target efficiently. Because of the low reaction temperature and high efficiency, RT-RPA enhances our ability to detect diverse pathogens.

Since the outbreak of COVID-19, researchers have made full use of RT-RPA to develop SARS-CoV-2 detection methods. They mostly combined SYBR Green I and/or lateral flow strips (LFS) with RT-RPA to construct the two-plex and visual detection platforms, but the amplification and detection were separate, which easily caused aerosol contamination and false-positive results. To address this problem, Liu D et al. (2021) integrated RT-RPA and a universal lateral flow (LF) dipstick detection system into a single microfluidic chip. The transfer of the RT-RPA mixture to the lateral flow strip is simple, and the chip device is inverted and simply shaken with no valving. To incorporate sample preparation, Tang et al. (2022) also developed a rapid integrated-RPA (I-RPA) system to detect SARS-CoV-2, which comprises a cartridge and an automatic nucleic acid detection device. No additional nucleic acid extraction processes are needed, so the whole time from adding the raw sample to obtaining the result is only 30 min. Moreover, to specifically detect the amplification products and amplify the signal, the CRISPR/Cas system is regarded as the ideal strategy. Lopez-Valls et al. (2022) combined RT-LAMP with CRISPR/Cas13 and gold nanoparticles (AuNPs) to establish a SARS-CoV-2 detection platform called CRISPR/CAS-based colorimetric nucleic acid detection (CASCADE). Upon target recognition, Cas13a cleaves ssRNA oligonucleotides conjugated to AuNPs, thus inducing their colloidal aggregation, which can be easily visualized. In comparison, Guo et al. (2020) also presented a simple viral detection platform, CASdetec (CRISPR-assisted detection), to integrate Cas12b with RT-RAA (a modified version of RPA) for COVID-19 diagnosis. They executed the RT-RAA reaction within the tube while keeping the CDetection reagents within the cap of the tube for 30 min, after which the CDetection reagents were spun down into the tube for nucleic acid detection. Hence, a constant operating temperature near 37°C certainly has distinctive advantages over PCR-based viral detection.

Nevertheless, the weakness is that RT-RPA requires several proteins in the reaction, so it is not easy to directly integrate with other detection systems to develop a one-pot reaction. They often need to rely on specially designed tubes or microfluidic chips to realize physical isolation and mixing. Moreover, due to the smaller number of primers, RT-RPA often suffers from lower specificity than RT-LAMP. However, even so, low reaction temperature, lyophilized pellet format and high convenience make it popular in point-of-care testing (POCT) scenes. To clearly display the detection performance, we prepared Table 1 to compare several isothermal amplification-based methods, including both domestic and foreign approved kits.

**TABLE 1:** Comparison of several isothermal amplification-based methods developed for SARS-CoV-2 detection.

Name of assay	Principle	Company	Target gene	Sensitivity	Time (min)	References
RT-LAMP assay	RT-LAMP	—	N	100 copies/rxn	30	Baek et al., 2020
mRT-LAMP-LFB	RT-LAMP	—	ORF1ab, N	12 copies/rxn	40	Zhu et al., 2020
COVID-19-LAMP	RT-LAMP	—	ORF3a, E	42 copies/rxn	60	Chow et al., 2020
Suitcase lab	RT-RPA	—	E, N	15 copies/rxn	15	El Wahed et al., 2021
—	RT-RPA	—	N	8 copies/rxn	20	Behrmann et al., 2020
Respiratory Virus Nucleic Acid Detection Kit	RT-LAMP	CapitalBio Technology	ORF1ab, N	150 copies/ml	90	CapitalBio coporation, 2022
Novel Coronavirus (2019-nCoV) Nucleic Acid Detection Kit	CPA	Ustar Biotechnologies	ORF1ab, N	200 copies/ml	80	USTAR, 2022
iAMP COVID-19 Detection Kit	RT-LAMP	Atila BioSystems	ORF1ab, N	4 copies/μl	<90	Rai et al., 2021
ID NOW COVID-19	NEAR	Abbott Diagnostics Scarborough	RdRp	125 copies/ml	15	Abbott Diagnostics, 2020
SHERLOCK CRISPR SARS-CoV-2 Kit	RT-LAMP, CRISPR-Cas13	Sherlock Biosciences	ORF1ab, N	4.5 copies/μl–ORF1ab; 0.9 copies/μl–N	60	Sherlock Biosciences, 2021
SARS-CoV-2 DETECTR Reagent Kit	RT-LAMP, CRISPR-Cas12	Mammoth Biosciences	E, N	10 copies/μl	45	Broughton et al., 2020

CPA, cross priming amplification; NEAR, nicking enzyme amplification reaction.

**TABLE 2:** Comparison of different SARS-CoV-2 nucleic acid detection methods.

Methods	Sample types	Absolute quantification	Sophisticated instrument	Time	LOD	Target gene	Advantages	Disadvantages
Sequencing	Nasopharyngeal swab	N	Y	2–3 days	—	—	More precise, genomic profiling, new mutations detection	Time-consuming, expensive, specialized operators
RT-PCR	Nasopharyngeal swab, sputum, bronchoalveolar lavage fluid, stool	Y	Y	70–120 min	100 copies/ml	ORF1ab, N	Mature technology, complete supporting reagents, absolute quantification, low cost	Thermal cycling, specialized operators, time-consuming
RT-LAMP	Nasopharyngeal swab, sputum, stool	N	N	30–60 min	1 copies/μl	ORF1ab, N	Isothermal, simple, rapid, highly sensitive	Non-specific amplification, too many primers, ladder band
RT-RPA	Nasopharyngeal swab, sputum, stool	N	N	20–40 min	0.25–2.5 copies/μl	N	Isothermal, simple, rapid, highly sensitive	Non-specific amplification, no primer design software, too many enzymes in system
CRISPR-based assay	Nasopharyngeal swab, bronchoalveolar lavage fluid	N	N	30–60 min	0.9–10 copies/μl	ORF1ab, N, E	Isothermal, amplify the signal, easy to combine with isothermal amplification	Immature technology, reaction system to be optimized
Biosensors and microfluidics	Nasopharyngeal swab, sputum, bronchoalveolar lavage fluid	N	N	60 min	0.5 copies/μl	N, M	Miniaturization, simple, real-time detection	Sensitive to surrounding environment, complex design, high cost

## Clustered regularly interspaced short palindromic repeat-based diagnostic methods

The CRISPR (clustered regularly interspaced short palindromic repeat) system is an acquired immune system that exists in bacteria and archaea and is used to resist foreign invading bacteriophages or viruses (Broughton et al., 2020). In recent years, a variety of CRISPR-based nucleic acid detection technologies have been developed, represented by SHERLOCK (specific high-sensitivity enzymatic reporter unlocking), HOLMES (a 1-h low-cost multipurpose highly efficient system), and DETECTR (DNA endonuclease-targeted CRISPR transreporter), which are known as “next-generation molecular diagnostic technologies” (Safiabadi Tali et al., 2021). They mainly rely on the trans-cleavage activity of Cas proteins. When Cas proteins (Cas13a or Cas12a) bind to crRNA to form binary complexes, they scan the target RNA or DNA, recognize the protospacer adjacent motif (PAM) sequence, and form Cas13a-crRNA-ssRNA or Cas12a-crRNA-dsDNA ternary complexes (Abudayyeh et al., 2016; Li et al., 2018). Next, the ternary complexes will activate the *cis*-cleavage activity of Cas proteins (Cas13a or Cas12a) and specifically cleave the target RNA or DNA and then induce powerful trans-cleavage activity to non-specifically cleave ssRNA or ssDNA. Therefore, as long as we add the single-stranded fluorescent reporter (ssRNA or ssDNA reporter) into the system, the results can be visualized and amplified.

The ability to amplify the signal is the basis for highly sensitive CRISPR-mediated nucleic acid detection. For SARS-CoV-2 detection, most of the current CRISPR-based methods briefly include the following three steps (Broughton et al., 2020; Joung et al., 2020; Patchsung et al., 2020): (1) isothermal amplification of the sample for less than 60 min, such as RT-LAMP, RT-RPA, RCA, or NASBA; (2) CRISPR-based detection of the amplified SARS-CoV-2 RNA after incubation for approximately 30 min; and (3) a lateral flow assay for displaying the results after 2 min of incubation. The SherlockTM CRISPR SARS-CoV-2 kit is the first FDA-authorized CRISPR-based diagnostic test for viral RNA detection (Joung et al., 2020). Nevertheless, the separation of amplification and detection easily causes aerosol contamination, which prompted Wang et al. (2021) to develop a one-pot visual reverse transcription (RT)-LAMP-CRISPR (opvCRISPR) method for SARS-CoV-2 detection. The RT-LAMP reagents are incubated at the bottom of the tube, while the CRISPR/Cas12a reagents are added to the lid. SARS-CoV-2 RNA templates are amplified by RT-LAMP, followed by mixing with Cas12a reagents for cleavage. In comparison, Ding et al. (2020) provided a true single reaction system named AIOD-CRISPR (All-In-One Dual CRISPR-Cas12a) for visual SARS-CoV-2 detection (Zhang et al., 2021). The components for both RT-RPA and CRISPR-12a were prepared in one pot, completely circumventing the separate preamplification of the target RNA or physical separation of Cas proteins. Furthermore, to realize point-of-care testing (POCT), de Puig et al. (2021) developed a minimally instrumented SHERLOCK (miSHERLOCK) device that combines built-in sample preparation from saliva, room temperature stable reagents, battery-powered incubation, and mobile phone-enabled results interpretation. Unlike Cas12/Cas13-based platforms, Cas9 is reported not to produce trans-cleavage activity on substrates, so it is not suitable for trans-cleavage signal output. To circumvent this, Azhar et al. (2021) constructed a platform called the FnCas9 Editor Linked Uniform Detection Assay (FELUDA) for COVID-19 diagnosis. They designed FELUDA as a direct, non-cleavage, affinity-based method of detection, working with single nucleotide mismatch sensitivity.

In summary, emerging CRISPR technologies have been widely applied in combination with isothermal amplifications for SARS-CoV-2 detection. Most of the methods regard it as a downstream means of amplifying the signal and further improving the sensitivity (Zhang et al., 2021). However, Cas proteins need to recognize a certain PAM sequence, which is still a rate-limiting step for widespread applications. Meanwhile, the possible reasons why CRISPR-based commercial products are still very few are as follows: First, the system and technology of CRISPR itself is not as mature as PCR; Second, the auxiliary instruments and reagents are not complete; Third, the advantages over PCR, such as cost, stability, and convenience, are not obvious now. Therefore, the real transformation of CRISPR technology requires joint efforts from academia and industry.

## Biosensors and microfluidics

Microfluidics, such as microchannels, microchambers, and microdroplets, have the advantages of smaller reaction volumes, higher detection throughput, ease of integration, and portability compared to traditional detection methods. These characteristics endow microfluidics with the potential to become a powerful technology to meet nucleic acid detection demands. Currently, most microfluidic devices are made from polymer materials such as PDMS (polydimethylsiloxane) and PMMA (polymethyl methacrylate) (Chu et al., 2022). Centrifugal force is the most commonly used fluid manipulation method, followed by electrochemical pumping and capillary action.

With the progression of the COVID-19 pandemic, a variety of methods based on microfluidics have been developed. Combined with RT-PCR, Yang J. et al. (2021) presented a microfluidic cartridge-based sample-to-answer POC device adapted for SARS-CoV-2 detection directly from self-collected saliva specimens. Similarly, Ji et al. developed a centrifugal disc-direct RT-qPCR (dirRT-qPCR) assay for multiplex detection of SARS-CoV-2 and influenza A and B in pharyngeal swab samples in an automated manner (Ji et al., 2020). To displace the thermocycling process, microfluidic chips are also combined with isothermal amplification. Huang et al. (2021) provided a two-stage isothermal amplification method, which consists of a first-stage basic RPA and a second-stage fluorescence LAMP, as well as a microfluidic-chip-based portable system. Due to the ability to amplify the signal, emerging CRISPR has become another promising technology to combine with microfluidics. Chen et al. (2022c) developed a dual-CRISPR/Cas12a-assisted RT-RAA assay and a “sample-to-answer” centrifugal microfluidic platform that can automatically detect 1 copy/μl of SARS-CoV-2 within 30 min. To further fulfill the public health need for a clinically relevant surveillance technology that detects multiple pathogens quickly, Welch et al. (2022) combined the CRISPR/Cas13 system and microfluidics to establish a multiplex detection platform called microfluidic Combinatorial Arrayed Reactions for Multiplexed Evaluation of Nucleic acids (mCARMEN), which can simultaneously detect 21 viruses, including SARS-CoV-2, other coronaviruses, and influenza viruses.

However, all the aforementioned methods need the extraction and amplification of nucleic acids first, which is a complicated process and prolongs the overall detection time. Therefore, Chu et al. proposed an extraction-free, amplification-free, and ultrasensitive fluorescence-based SARS-CoV-2 detection method based on a nanomaterial hybrid microfluidic biochip including 15 parallel sensing units (Chu et al., 2022). The high signal-to-noise ratio of the biochip and the high-precision laser scanner enables accurate detection of target signals. Another method based on molecular nanostructures and automated microfluidics was developed by Zhao et al. (2021) and named after an electrochemical system integrating reconfigurable enzyme-DNA nanostructures (eSIREN). It leverages a molecular circuitry comprising catalytic enzyme-DNA nanostructures to directly recognize target RNA and automated microfluidics to interface the molecular circuitry with the embedded electrodes to transduce the direct target recognition into an amplified electrical signal.

In addition to the literature, many companies have developed point-of-care (POC) testing systems for SARS-CoV-2 detection (Yang J. et al., 2021). For example, the Cepheid Xpert Xpress SARS-CoV-2/Flu/RSV system (CA, USA) can provide rapid detection of SARS-CoV-2 in 25 min and provide results for four pathogens in 36 min, with less than 1 min hands-on time. Moreover, the Abbott’s ID NOWTM system (IL, USA) adopts isothermal amplification technology, and it can process nasopharyngeal swabs and saliva samples and provide results in 15 min (Abbott, 2022). Although many POC devices for SARS-CoV-2 detection have been reported, there are several limitations, such as high cost and low throughput; furthermore, sample preparation cannot be easily incorporated into the detection process.

Overall, although research in microfluidics has been advancing for almost a half-century, its adoption into real-world applications has been slow and has encountered hurdles. The reasons include immature core technologies, poor compatibility of materials with biomolecules, difficulty in integration with peripheral devices, high production cost, and insufficient multidisciplinary talent. In recent decades, with advancements in material science and microfluidic device manufacturing techniques, great developments in microfluidics in diagnostics have been achieved.

## The influences of severe acute respiratory syndrome coronavirus 2 variants on diagnostics

According to the level of virulence and risk, the World Health Organization (WHO) classified SARS-CoV-2 variants into variants of concern (VOCs), variants of interest (VOIs), and alerts for further monitoring (AFM) (World Health Organization, 2022a). VOCs include alpha, beta, gamma, delta, and micron variants, which are more infectious and can cause severe diseases with increased immune escape. The emergence of the variants makes it difficult to diagnose and reduce the effectiveness of treatments and vaccines. At present, the Omicron variant is the global-dominated strain with multiple subvariants. The Omicron variant (B.1.1.529) possesses more than 30 mutations and at least 15 amino acid changes in the receptor binding domain (RBD), which is a key structure for invasion (Barnes et al., 2020; Cui et al., 2022). It would be 2.8 times more infectious than the Delta variant, mainly because of RBD mutations N440K, T478K, and N501Y (Chen et al., 2022a). Currently, the Omicron variant BA.2 is the main focus of attention due to its widespread use in Europe, America, and China, with increased transmissibility and immune escape in people. Compared to BA.1, the effective reproduction number of the Omicron variant BA.2 is 1.4-fold higher (Yamasoba et al., 2022). A timely and effective diagnosis would cut the transmission chain and reduce excess mortality raised by overhigh transmissibility. However, variants pose new challenges to current detection methods, as shown below.

First, the accuracy of commonly used target genes becomes less reliable. Double-target (ORF1ab and N gene) reagents are widely used in the detection of SARS-CoV-2, while many mutations of the Omicron variant exist in the N gene, triggering false negative results in the detection (Yu et al., 2020; Cui et al., 2022). Similar inaccuracy of the target gene also occurs in other SARS-CoV-2 variants. For example, Chen et al. (2022b) evaluated the influence of VOCs on commercial kits, suggesting that beta and delta variants adversely affected the sensitivity of ORF1ab gene analysis, and N gene analysis completely failed in the gamma variant. Therefore, the existing detection methods should be modified to detect new variants. More conserved mutations for the test should be considered, and mutations in the S gene are also suitable candidates. For the Omicron variant, the community track platform in Denmark developed and implemented the RT-PCR test using the L452R mutation, with an estimated specificity of 99.99% based on retrospective analysis (Spiess et al., 2021). Niu et al. (2022) developed a PCR-based CRISPR/Cas13a detection system (PCR-CRISPR) to improve the sensitivity and portability of SARS-CoV-2 HV69-70del mutant site detection.

Second, the mutations could limit the efficacy and sensitivity of the test. In the assay kit of Sansure Biotech (Changsha, China), the gamma variant affected the PCR amplification efficiency of ORF1ab (Chen et al., 2022b). Rajib et al. (2022) also reported that the Delta variant containing a mutation in the probe binding region of the E gene exhibited atypical PCR amplification and might induce false negative results. To improve the sensitivity and specificity, Liang et al. (2022) combined PCR with CRISPR technology to develop a CRISPR-Cas12a-based assay to detect and trace Omicron variants. They designed two sets of specific crRNAs based on Omicron mutations, including crRNA-S-37X (covering S371L, S373P, and S375F) and crRNA-S-49X (covering Q493R, G496S, and Q498R). Artificial introduction of additional mutations around the target mutation site into crRNAs could significantly improve identification efficacy. This quick test could be routinely implemented in resource-limited conditions to monitor and track the spread of Omicron variants.

**FIGURE 3:**
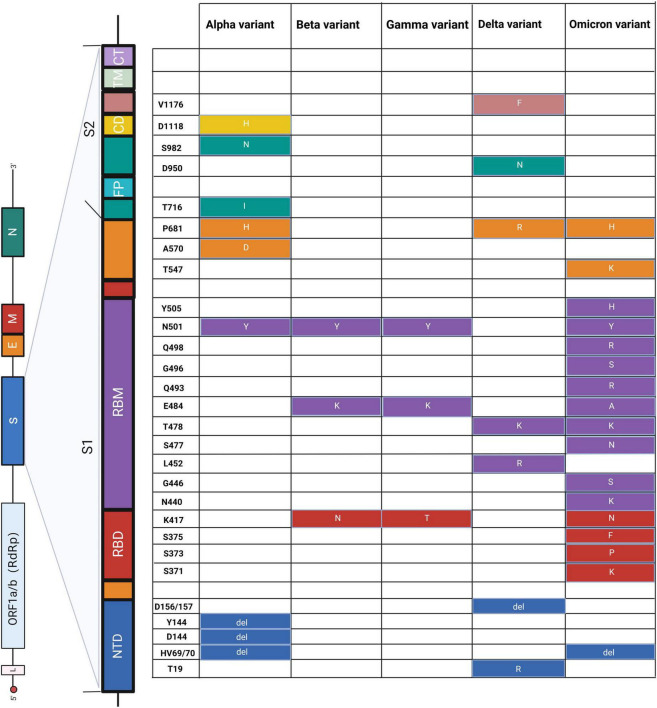
Schematic diagram of amino acid mutations in the S gene of SARS-CoV-2. The **left panel** shows the genome of SARS-CoV-2 and the detailed structure of the S gene. The S1 and S2 subunits and a transmembrane domain constitute the spike protein (S). RBD and RBM bind to the host cell receptor. S2 consists of FP, CD, and CT, contributing to membrane fusion. Substitutes of amino acids of mutations are presented on the **right panel**, and the colors correspond to the structure of the S gene. NTD, N-terminal domain; RBD, receptor-binding domain; RBM, receptor-binding motif; FP, fusion peptide; CD, connecting domain; TM, transmembrane domain; CT, cytoplasmic tail. Alpha (Berenger et al., 2021; Borsova et al., 2021; Hale et al., 2021; Fu et al., 2022; Oh et al., 2022; Ratcliff et al., 2022), beta (Hale et al., 2021; Dikdan et al., 2022; Fu et al., 2022), gamma (Hale et al., 2021; Ratcliff et al., 2022), delta (Berenger et al., 2021; Hale et al., 2021; Norz et al., 2021; Oh et al., 2022; Rosato et al., 2022), and omicron (Bloemen et al., 2022; Dachert et al., 2022; Rasmussen et al., 2022).

On the other side of a double-edged sword, we can utilize these significant mutations to distinguish different SARS-CoV-2 variants (Figure 3). For example, Hale et al. (2021) used PCR to effectively distinguish alpha (HV69/70, N501Y), beta (N501Y, K417N, E484K, P681R), gamma (N501Y, E484K), and delta (P681R) variants through mutations HV69/70, N501Y, K417N, and E484K on the S gene, which showed 100% specificity and sensitivity. Similarly, Liang et al. (2021) developed a reliable and fast CRISPR-Cas12a system based on PCR to successfully utilize S gene mutations (K417N/T, L452R/Q, T478K, E484K/Q, N501Y, and D614G) to distinguish alpha, beta, gamma, and delta variants. This CRISPR-based approach can be used to screen emerging mutations and is immediately implemented in laboratories where nucleic acid tests are already performed or in resource-limited settings. Moreover, multiplex detection in a single reaction is a significant research direction for simultaneously monitoring multiple mutations and variants, in contrast to traditional tests.

## Discussion and prospects

The COVID-19 pandemic highlights the need for diagnostic methods that can be rapidly adapted and deployed in a variety of settings. In this review, various SARS-CoV-2 nucleic acid detection methods, including sequencing, RT-PCR, isothermal amplification (LAMP and RPA), CRISPR, and biosensors, have been summarized and evaluated individually. To further clarify the strengths and weaknesses, we prepared a table to compare the above methods (Table 2). No method is perfect, and we need to choose a suitable method according to the specific application scenario and purpose.

**FIGURE 4:**
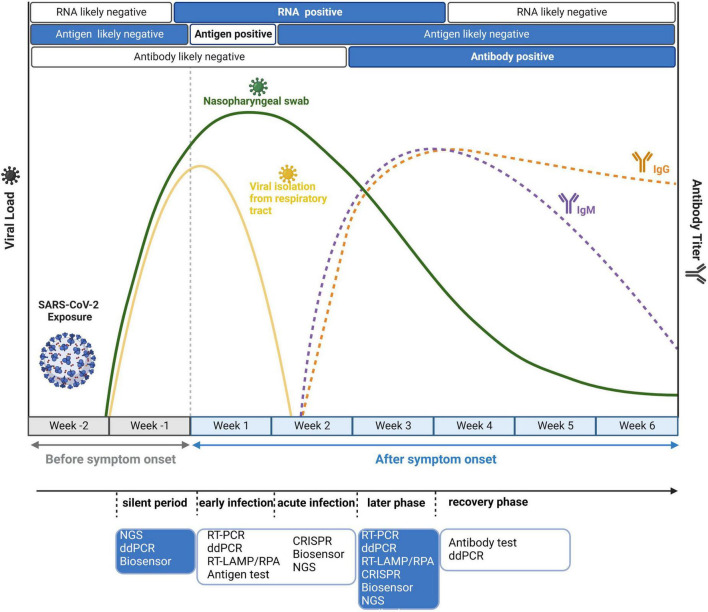
Disease dynamics and optional diagnostic tests for COVID-19. Because of virus load and antibody dynamics, it is necessary to adopt different detection strategies at different time points. Before symptom onset, the optimal choice is nucleic acid testing because its limit of detection (LOD) can reach 10^2^–10^3^ copies/ml. After symptom onset, antigen tests and viral RNA detection are all feasible in the first week, while antibody tests are positive later in the disease course.

Although these methods are robust and sensitive, their practical effect would be modified by different stages of individual-specific immune reactions and burdens of the virus. Therefore, it is important to adopt suitable detection strategies at different time points after infection. As shown in Figure 4, we also mark the distinct time points for antigen and antibody detection apart from nucleic acid detection. Because of the long incubation period of SARS-CoV-2, it is advisable to adopt several detection strategies. Based on the timeline, in the silent period of infection, most detection methods could not detect SARS-CoV-2 due to the very low virus load. In this case, NGS could realize real-time surveillance, while NGS is not suitable for large-scale screening. ddPCR is highly sensitive and suitable for detecting low viral load specimens that interfere with community transmission in the early stage of the epidemic (Dong et al., 2021). Suo et al. (2020) simultaneously compared the limit of detection (LOD) between ddPCR and RT-PCR and found that the LOD of ddPCR was 2.1 copies/reaction (ORF1ab gene) and 1.7 copies/reaction (N gene), much lower than that of RT-PCR (1,039 and 873.2 copies/reaction). Moreover, the combination of biosensors and rapid antigen testing can also be applied to detect asymptomatic infected patients, and the specificity is over 99% (Schuit et al., 2021). After symptom onset, both antigens and nucleic acids are suitable. Primary screening could use antigen testing, which is cost-effective and fast. Considering the high false positive rate of antigen detection, antigen testing alone is not recommended. Other nucleic acid tests, such as RT-PCR, RT-LAMP, and CRISPR, should be employed to further confirm the diagnosis. In the early, acute and later infection stages, nucleic acid testing can be used as the diagnostic choice for COVID-19. However, in the recovery period of infection, antibody testing is much more reliable. IgM is expressed 3–7 days after infection and can be detected in the second week after infection, whereas IgG antibody needs 8 days to reach a detectable level (Peeling et al., 2022). Overall, previous studies suggest that the right test at the right time is the key to correct the diagnosis of COVID-19.

False negative and false positive results have always been a concern for nucleic acid testing. Although the sensitivity and specificity of current detection methods such as RT-PCR have almost reached approximately 100% in diagnosing COVID-19, cases of false negatives and positives are continuously reported (Chan W. et al., 2020; Pokhrel et al., 2020; Kanji et al., 2021). Problems in every step from sample preparation to detection could influence the results, probably contributing to the false results. We summarized the possible reasons in Table 3. Of note, as mentioned in the table, new mutations would decrease the accuracy and sensitivity of nucleic acid testing from different aspects, especially the mutations in primers and the target region of the probe, giving rise to false negative results. Furthermore, the dynamics of virus load during infection would extend the limit of detection (LOD) of testing. The LODs of RT-PCR are reported as 5–7,740 copies/ml, and the LODs of RT-LAMP are 2–304 copies/reaction (Yang S. et al., 2021). Other factors, including RNA extraction, efficiency of reverse transcription and settings of reaction, cannot be ignored.

**TABLE 3:** Possible reasons for false negative and positive results in nucleic acid detection.

	Factors	References
**Sample preparation**		
Sample collection	• Improper materials: the swap containing calcium alginate or a stick has inhibitors against PCR. • Source of sample: gastrointestinal symptoms are related to prolonged virus RNA in gastrointestinal when upper respiratory symptoms disappeared with undetected virus RNA in respiratory tract. • Time of sample: the longer the interval between symptoms and detection is, the higher the probability of false negative is.	Premraj et al., 2020; Natarajan et al., 2022
Sample store	• Improper store and transport sample: RNA might degrade due to inappropriate temperature.	
**Detection technology**		
Target gene	• Deletion of gene fragments and genome variation on target gene would affect the use of existing primers.	Su et al., 2020
Limiting of detection	• Reaction settings, amplification efficiency, reverse transcription efficiency, etc., could influence the range of limiting of detection.	Yang S. et al., 2021
**Patients**		
Drugs/Inhibitors	• heme and humic acid are common PCR inhibitors; drugs like Acyclovir also have been reported to inhibit Taq DNA polymerase	Yedidag et al., 1996
Infection dynamics and severity	• Infection severity and upper respiratory viral load of individual differences might contribute to false-negative results	

Last but not least, several pain points remain to be solved for current SARS-CoV-2 nucleic acid detection methods, which is also a future direction for development. First, integrated sample preparation is necessary, especially for POC diagnostic devices. Currently, most of the sample preparations are performed separately in the existing detection platforms, which increases the complexity of the test and reduces user friendliness. It is desirable to combine sample preparation with amplification and detection. Although some studies have developed sample preparation-free methods by utilizing RT-PCR and RT-LAMP, there is still a long way to go to improve the stability, sensitivity, throughput, and compatibility. A fully simplified process (raw sample-in-answer-out) is highly demanded.

Second, with the increasing number of targets and samples, nucleic acid detection raises higher requirements for detection throughput, multiplicity and portability. However, traditional RT-PCR and isothermal chips could hardly meet these requirements. Biochips and microfluidics can physically isolate different primer pairs through micropores or microchannels, enabling multiplex detection of targets in a closed space. For example, Xing et al. (2020) developed the RTisochipTM-W platform by combining isothermal amplification technology with a microfluidic disc chip, which can simultaneously detect 19 common viruses from 16 samples in a single run. Nevertheless, the design is actually to stack multiple chip devices together to realize high-throughput detection, and each instrument is expensive and not portable. Similarly, we also compared the international mainstream nucleic acid detection platforms based on microchips or microfluidics technology (Table 4) and found that they are basically portable and integrated. However, their detection throughput is also low, and only one sample can be detected at a time. To address this, Biofire has launched the FilmArray 2.0 platform, but in fact, it is also a combination of multiple FilmArray 1.0 devices, similar to the aforementioned RTisochipTM-W platform. Overall, there is still a lack of a detection platform simultaneously integrated with high detection throughput, multiplicity and portability.

**TABLE 4:** Comparison of mainstream nucleic acid detection platforms.

Platforms	Company	Principle	Volume (cm)	Weight (kg)	Throughput	Target	Time (min)
FilmArray	Biofire	Nested PCR	39.3 × 25.4 × 16.5	7.3	1	24	60
GeneXpert Omni	Cepheid	Nested qPCR	8 × 11 × 23	1	1	1–2	30–60
Io system	Atlas Genetics	PCR	27.7 × 27.5 × 38.4	10	1	24	30
Cobas Liat	IQuum	qPCR	19 × 11.4 × 24.1	3.76	1	1–2	20
ID Now	Abbott Molecular	NEAR	21 × 15 × 19	3	1	1–2	5–13
RTisochip-W system	CapitalBio	NASBA	58.6 × 69.0 × (17.5 × 4)	33 × 4	16	19	90
iPonatic	Sansure Biotech	qPCR	–	<10	1	1–2	15–45
Easy NET	USTAR Biotech	CPA	26 × 37 × 52	13	2	1–2	80–90

NEAR, nicking enzyme amplification reaction; CPA, crossing priming amplification.

Third, with the rapid spread of the COVID-19 pandemic, another major focus of future work is to develop POC and home tests that do not require specialized operations. In this way, people can acquire the detection results quickly and seek professional help instead of waiting longer for results from the laboratory. To increase the detection throughput and extensibility, POC tests can be combined with the automated sample processing system, allowing patients to be diagnosed sooner. Moreover, we should also develop more novel biosensors as POC diagnostic devices for SARS-CoV-2 detection, such as electrochemical sensors, field-effect transistor (FET)-based sensors, magnetic biosensors, enzyme-based sensors, and DNA biosensors. It is believed that through our joint efforts, nucleic acid detection methods will continue to innovate to better meet the detection needs of infectious diseases.

## Author contributions

YZ and MZ contributed to the central idea and coordinated the writing of the manuscript. ZJ and ST read, discussed, and revised the manuscript. All authors contributed to the article and approved the submitted version.

## Footnotes

1
https://covid19.who.int/
